# Marijuana-Induced Lung Injury: A Case Report and a Review of the Literature

**DOI:** 10.7759/cureus.34635

**Published:** 2023-02-04

**Authors:** Alexander T Phan, Janie Hu, Henrik H Ghantarchyan, Viet-Tien P Nguyen, Mufadda Hasan

**Affiliations:** 1 Internal Medicine, Arrowhead Regional Medical Center, Colton, USA; 2 Pulmonary and Critical Care Medicine, Arrowhead Regional Medical Center, Colton, USA

**Keywords:** tetrahydrocannabinol, drug-induced lung injury, pulmonology, pneumonitis, marijuana

## Abstract

Marijuana is a commonly abused illicit substance around the world, and lung injury related to its use has seldom been cited in the literature. Most cases describe marijuana-induced lung injury via vaping and the use of butane hash oil; however, no cases, to our knowledge, have associated lung injury related to marijuana smoke in the form of rolled "blunts" or cigarettes. We describe the case of a patient who presented to the hospital due to chest computed tomography findings demonstrating diffuse bilateral opacifications without signs of systemic inflammatory response syndrome. Bronchoscopy with bronchoalveolar lavage and sputum cultures failed to identify an infectious etiology, and serologies were negative for autoimmune etiologies. We aim to contribute to the limited body of literature describing marijuana-induced lung injury.

## Introduction

Marijuana is currently the most commonly abused illicit substance in the world. Marijuana is a term that refers to parts of the plant *Cannabis sativa* that contain large concentrations of tetrahydrocannabinol (THC). THC is the molecular compound that is responsible for producing changes in mental states in users. Marijuana, also commonly referred to simply as 'cannabis', is commonly abused worldwide and is easily accessible given its recent legalization in several states [1]. Marijuana is commonly inhaled (smoking, dabbing, vaping) and, as such, can increase one's risk for lung injury.

Drug-induced lung injury (DLI) is defined as lung injury associated with the use of pharmacologic agents, such as medications, drugs of abuse, and herbal medicines. DLI occurs when there is an adverse drug reaction that involves the bronchi, lung parenchyma, pleura, and pulmonary vessels. Consequently, the pathologies that may arise as a consequence of DLIs are vast. The temporal relationship between drug usage and lung injury is also variable, ranging from minutes to years; however, it is most commonly on the order of weeks to months. Risk factors for developing DLI include advanced age, existing pulmonary disease, previous radiation exposure to the lung, and renal impairment [2].

The pathogenesis of DLI is unclear, though two likely mechanisms have been proposed. The first mechanism is linked to the use of cytotoxic agents, as these drugs may damage type I pneumocytes, airway epithelial cells, and pulmonary vascular endothelial cells. The second mechanism proposes that the drug may act as a hapten, triggering an immune response that may initiate a cascade of effects that injure the lungs. In the literature, few studies have reported an association between marijuana/cannabis use and lung injury. Here, we present the case of marijuana-induced pneumonitis.

## Case presentation

**Figure 1:**
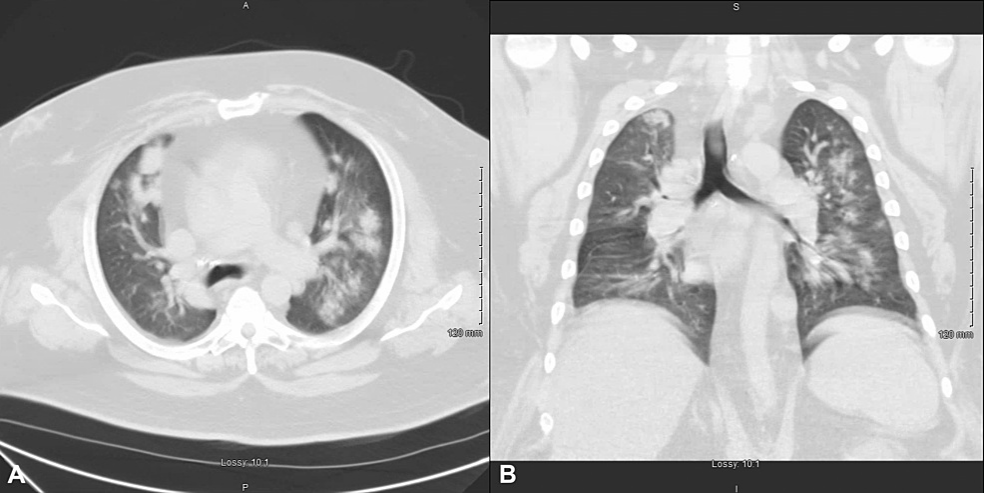
A) axial section of a chest computed tomography demonstrating bilateral patchy and nodular airspace infiltrates; B) coronal section of chest computed tomography demonstrating bilateral patchy airspace infiltrates

A 51-year-old male with a past medical history of hypertension, heart failure with preserved ejection fraction, severe obstructive sleep apnea, obesity, diabetes mellitus, and hypertension presented to the hospital at the urge of his pulmonologist due to shortness of breath of five days duration and abnormal findings on a chest computed tomography (CT) performed earlier that day. He reported a productive yellow, non-bloody cough. He denied nausea, vomiting, chest pain, abdominal pain, lower extremity edema, and pillow orthopnea. He reported compliance with his nightly bilevel-positive airway pressure ventilation mask. Social history was notable for over 20 years of smoking marijuana in the form of a "blunt" on a daily basis, but denied ingesting edible forms or vaping of any form. The patient also denied alcohol use, tobacco use, and all other drug use. His home medications included oral aspirin 81mg daily, oral simvastatin 20mg daily, oral metformin 1000mg twice daily, oral glipizide 10mg twice daily, oral sitagliptin 100mg daily, oral furosemide 40mg as needed, oral enalapril 20mg twice daily, and oral carvedilol 25mg twice daily. Initial vital signs: temperature of 98.9 F, blood pressure of 165/95 mmHg, pulse rate of 91, respiratory rate of 20, and oxygen saturation of 96% on 3L low flow supplemental oxygen. Physical exam was unremarkable except for bilateral wheezing and large body habitus. A chest CT obtained earlier in the day demonstrated bilateral patchy and nodular airspace infiltrates and cardiomegaly (Figure 1).

Initial laboratory studies demonstrated normal blood counts, mild hypokalemia, and elevated erythrocyte sedimentation rate (Table 1). An arterial blood gas on ambient air demonstrated mild alkalemia and hypoxemia without hypercapnia (Table 2). The patient was started on an antibiotic regimen of intravenous ceftriaxone 2g daily and oral azithromycin 500mg followed by 250mg daily due to concern for possible pneumonia. Additionally, he was administered inhaled albuterol due to wheezing noted on examination. He was also restarted on his home medications for his other chronic illnesses, except for diabetes mellitus, which was managed with insulin in the inpatient setting. The pulmonology specialist was consulted and a bronchoscopy was recommended due to a prior history of recurrent pneumonitis. The subsequent day, a bronchoscopy with bronchoalveolar lavage (BAL) of the lingular lobe was performed. The bronchoscopy revealed narrowing of the right middle lobe opening secondary to edema, but was otherwise unremarkable. The BAL cytology demonstrated moderate cellularity composed of alveolar macrophages and bronchial epithelial cells, and it was also negative for malignant cells. The BAL cell count and differential demonstrated elevated lymphocytes and bronchial epithelial cells (Table 3). BAL testing for respiratory syncytial virus, cytomegalovirus, herpes simplex virus, pneumocystis jirovecii, and acid fast bacilli were also negative.

**Table 1:** Initial serum laboratory studies demonstrating mild hypokalemia μL - microliter, g - gram, dL - deciliter, fL - femtoliter, mEq - milliequivalent, L - liter, mmol - millimole, mg - milligram, BUN - blood urea nitrogen, ESR - erythrocyte sedimentation rate, mm - millimeter, hr - hour

Laboratory test	Reference values	Measured values
White blood cells (cells/μL)	4,300-11,100	10,800
Hemoglobin (g/dL)	11.5-15.5	13.4
Hematocrit (%)	36-46	41.6
Platelet (cells/μL)	120,000-360,000	310,000
Mean corpuscular volume (fL)	80-100	85
Sodium (mEq/L)	135-148	139
Potassium (mEq/L)	3.5-5.5	3.3
Chloride (mEq/L)	98-110	100
Bicarbonate (mmol/L)	24-34	25
BUN (mg/dL)	8-20	19
Creatinine (mg/dL)	0.5-1.5	0.8
ESR (mm/hr)	0-10	87

**Table 2:** Initial arterial blood gas on ambient air demonstrating mild alkalemia and hypoxemia mmHg - millimeters of mercury, mmol - millimole, L - liter

Laboratory test	Reference values	Measured values
Arterial pH	7.35-7.45	7.47
Arterial carbon dioxide (mmHg)	35-45	36
Arterial oxygen (mmHg)	75-100	51
Arterial bicarbonate (mmol/L)	22-26	26
Arterial oxyhemoglobin (mmol/L)	94-97	83.5
Carboxyhemoglobin (%)	0-1.5	1.4

**Table 3:** Bronchoalveolar lavage cell fractions demonstrating elevated lymphocyte cell fraction, elevated bronchial epithelial cells, and reduced macrophage cell fraction

Laboratory test	Reference values	Measured values
Neutrophils (%)	2-11	2
Lymphocytes (%)	2-15	20
Eosinophils (%)	0-1	1
Macrophages (%)	60-80	50
Bronchial epithelial cells (%)	3-21	33

Urine legionella antigen, urine streptococcus pneumoniae antigen, urine histoplasma antigen, and serum influenza types A and B polymerase chain reaction testing were within normal limits. Serum 1,3-beta-D-glucan testing and coccidiosis antibody testing were also negative. An echocardiogram was performed, demonstrating mild left ventricle wall thickening; the remainder of the exam did not demonstrate diastolic dysfunction, systolic dysfunction, or elevated right atrial pressures. Antinuclear antibody screen, anti-neutrophil cytoplasmic antibody (ANCA) screen, anti-proteinase-3 (c-ANCA) titer, myeloperoxidase antibody (p-ANCA) titer, and atypical p-ANCA titer were within normal limits. No systemic corticosteroids or disease-modifying agents were given to the patient. His symptoms resolved over the course of three days, and he was discharged from the hospital with instructions to discontinue marijuana use and follow up in the pulmonary medicine clinic.

**Figure 2:**
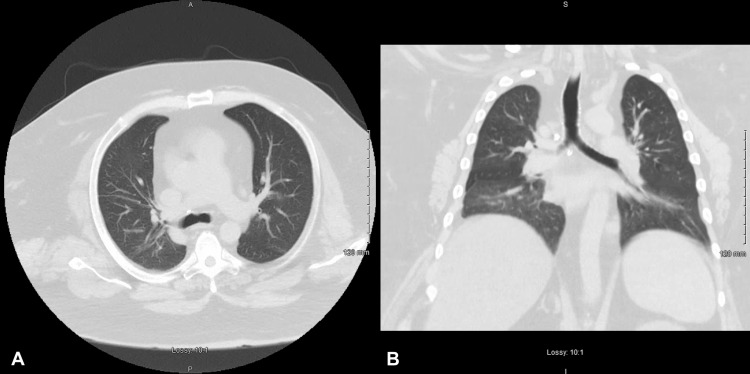
A) axial section of a chest computed tomography demonstrating interval resolution of bilateral pulmonary opacifications; B) coronal section of a chest computed tomography demonstrating interval resolution of bilateral pulmonary opacifications

At one month follow-up in the pulmonary medicine clinic after hospital discharge, the patient reported that he had successfully quit smoking marijuana completely and denied any symptoms of shortness of breath. The patient had a repeat chest CT performed at this time, which demonstrated a complete resolution of the previous findings (Figure 2). Spirometry was also performed, which did not demonstrate evidence of obstructive lung disease. Since then, the patient has completely abstained from smoking marijuana and has not had recurrent bouts of pneumonitis. He is currently alive and well at the four-year follow-up mark. Repeat urinalyses have not demonstrated positivity for tetrahydrocannabinol.

## Discussion

The diagnosis of drug-induced lung injury requires five criteria: 1) use a of a drug known to cause lung injury, 2) clinical manifestations have been reported to be induced by said drug, 3) other causes of clinical manifestations have been ruled out, 4) improvement of the clinical manifestations following drug discontinuation, and 5) exacerbation of the clinical manifestations after resuming drug administration [2]. In a study by Akira et al., chest computed tomographies of 60 patients with drug-induced pneumonitis were reviewed, demonstrating diffuse ground glass opacities in most cases. In a subset of patients with DLI secondary to exposure to herbal substances, patchy consolidation was also noted in addition to the diffuse ground glass opacities [3]. Marijuana is an herbal substance that has also been reported to induce lung injury, though reports are limited to case studies. Previous manuscripts have identified lung injury associated with inhalation of cannabis via vaping and butane hash oil use. Ground-glass opacities were noted in every case, and the distribution is typically diffuse, with some cases demonstrating diffuse alveolar infiltrates with patchy consolidation [4-8].

In our patient's case, we identified marijuana smoking as the etiology for his lung injury pattern based on a thorough diagnostic workup. Previous reports have described symptomatic marijuana-induced lung injury as a disease entity, satisfying criteria one and two. We ruled out autoimmune and infectious etiologies for the patient's clinical manifestations through an extensive workup as detailed in the case presentation, satisfying criterion three. Additionally, since the patient's symptoms resolved and never relapsed following discontinuation of marijuana smoking, criterion four is met. We are unable to satisfy criterion five, as it would have been unethical to administer a medication that has the potential to cause patient harm. One study by Yamauchi et al. examined the BAL cytology of patients with drug-induced pneumonitis. The study found that, in patients with DLI, the most common BAL finding was elevated lymphocyte cell fractions [9]. Consistent with this study, our patient's BAL cytology also showed an elevated lymphocyte cell fraction, suggestive of DLI. Interestingly, the chest CT of our patient demonstrates diffuse bilateral patchy opacifications and similarities resembling what He et al. describe as a "tree-in-bloom" [5]. This "tree-in-bloom" appearance may be a radiologic pattern that some patients with marijuana-induced lung injury exhibit and warrants further study.

We present a rare case of drug-induced lung injury due to marijuana smoking. Though it is rare, this case highlights the importance of a thorough social history during initial history taking. We hope that physicians consider marijuana smoking as a potential etiology of drug-induced lung injury after ruling out infectious processes and autoimmune etiologies. Future studies may investigate the underlying mechanism of marijuana-induced lung injury and better define the spectrum of radiologic patterns associated with said injury, as marijuana is commonly used around the world.

## Conclusions

Marijuana, also commonly referred to as cannabis, is a commonly abused illicit substance that has gained worldwide popularity. Due to its common use, we believe marijuana-induced lung injury is a diagnosis that may increase in prevalence in the coming years. Its ability to produce a significant disease burden to patients requires further studies as it has only been previously reported in case studies. Consistent with other studies, we identified elevated lymphocyte cell fractions in our BAL and noted patchy opacifications with a "tree-in-bloom" pattern on chest CT. Clinicians should consider marijuana-induced lung injury in patients presenting with diffuse pulmonary opacifications after ruling out infectious and autoimmune etiologies. We aim to increase awareness of this pulmonary pathology by describing a unique patient case and sharing our radiologic, bronchoscopic, and laboratory findings.
